# Autochthonous Starter Cultures Are Able to Reduce Biogenic Amines in a Traditional Portuguese Smoked Fermented Sausage

**DOI:** 10.3390/microorganisms8050686

**Published:** 2020-05-08

**Authors:** Igor Dias, Marta Laranjo, Maria Eduarda Potes, Ana Cristina Agulheiro-Santos, Sara Ricardo-Rodrigues, Ana Rita Fialho, Joana Véstia, Maria João Fraqueza, Margarida Oliveira, Miguel Elias

**Affiliations:** 1 MED—Mediterranean Institute for Agriculture, Environment and Development, IIFA-Instituto de Investigação e Formação Avançada, Universidade de Évora, Pólo da Mitra, Ap. 94, 7006-554 Évora, Portugal; mlaranjo@uevora.pt (M.L.); mep@uevora.pt (M.E.P.); acsantos@uevora.pt (A.C.A.-S.); sirr@uevora.pt (S.R.-R.); rita.b.fialho@gmail.com (A.R.F.); joana.vestia@gmail.com (J.V.); elias@uevora.pt (M.E.); 2 CIEQV—Life Quality Research Centre, Avenida Dr. Mário Soares n° 110, 2040-413 Rio Maior, Portugal; margarida.oliveira@esa.ipsantarem.pt; 3 ESAS, UIIPS—Instituto Politécnico de Santarém, Quinta do Galinheiro, S. Pedro, 1001-904 Santarém, Portugal; 4 Departamento de Medicina Veterinária, Escola de Ciências e Tecnologia, Universidade de Évora, Pólo da Mitra, Ap. 94, 7006-554 Évora, Portugal; 5 Departamento de Fitotecnia, Escola de Ciências e Tecnologia, Universidade de Évora, Pólo da Mitra, Ap. 94, 7006-554 Évora, Portugal; 6 CIISA—Centro de Investigação Interdisciplinar em Sanidade Animal, Faculdade de Medicina Veterinária, Universidade de Lisboa, Avenida da Universidade Técnica, 1300-477 Lisboa, Portugal; mjoaofraqueza@fmv.ulisboa.pt; 7 LEAF—Linking Landscape, Environment, Agriculture and Food, Instituto Superior de Agronomia, Universidade de Lisboa, Tapada da Ajuda, 1349-017 Lisboa, Portugal

**Keywords:** smoked fermented sausages, *Painho da Beira Baixa*, starter cultures, biogenic amines, food safety, food quality

## Abstract

Traditional smoked fermented sausages are highly appreciated in Portugal and are mostly manufactured according to traditional procedures. The aim of the present work was to evaluate the effect of autochthonous starter cultures on the safety and quality of a smoked fermented sausage, *Painho da Beira Baixa* (*PBB*), preserving its sensory quality. Physicochemical parameters, namely pH and water activity (a_W_), microbiological parameters, biogenic amines, colour, texture profile and sensory attributes were assessed. Different starters were selected based on our previous work. *Staphylococcus equorum* S2M7, *Staphylococcus xylosus* CECT7057, *Lactobacillus sakei* CV3C2, *Lactobacillus sakei* CECT7056 and a yeast strain (2RB4) were co-inoculated in meat batters at defined concentrations. Starters had a significant effect on the reduction of pH. Enterobacteria and *Listeria monocytogenes* were not detected in inoculated end-product sausages. Moreover, sausages inoculated with *S. equorum* S2M7/*L. sakei* CV3C2/yeast 2RB4 showed a significant reduction in the total content of biogenic amines. No significant differences between treatments were observed for colour and texture parameters, except for adhesiveness. The studied starters did not compromise the sensory characteristics of *PBB*. To our knowledge, this is the first comprehensive study on the quality and safety of this type of smoked fermented sausage from the central region of Portugal.

## 1. Introduction

Meat processing extends the shelf-life of raw materials, thus valorising them and increasing their economic value. In Portugal, Beira Baixa is one of the most important regions for the production of dry-cured ham, but also of smoked fermented sausages, where products like *Painho* stand out for their unique organoleptic characteristics. These result from the manufacturing know-how of generations of sausage producers and small processing units, according to traditional practices specific to each geographical area.

Generally, dry-fermented meat sausages are stable products in which sanitary risks are reduced thanks to five main aspects: pH reduction (mainly due to the microbial fermentation of carbohydrates); a decrease in water activity throughout the curing process; the addition of nitrates and nitrites (with antimicrobial properties that prevent and/or eliminate pathogenic and deteriorating microorganisms); the eventual use of starters with antimicrobial activity; and smoking, when applied (for the bacteriostatic and bactericidal effects of smoke). However, the onset of emerging pathogens, which pose new safety concerns, and the pressure to reduce additives, due to health issues, have stressed the need to study the microbiota of processing environments. Furthermore, the need for a general improvement in process efficiency has also determined the introduction of innovative processes in meat industries. The introduction of starters, together with the rigorous control of all conditions throughout the curing process, are among the main tools adopted by meat industries to improve the sanitary, nutritional, and sensory qualities. These tools may result in further advantages to the technological process, such as a higher degree of compliance and the shelf-life extension of sausages [1,2,3]. In Portugal, the use of starters is not common in micro and small processing units. When used, the available starters are not adapted to our processes and meat products, with consequent changes in sensory attributes of regional smoked fermented meat sausages. Therefore, the selection of autochthonous strains, more adapted to these meat products, is necessary. In fact, starter cultures are pure (individual) or mixed microbiological cultures of known microorganisms, inoculated at defined concentrations [4], that may promote and conduct the fermentation of sausages [5]. They significantly contribute to the quality and safety of meat products, mainly through their bioprotective (inhibition of foodborne pathogens growth) and fermentative/probiotic (production of secondary metabolites) actions [6,7,8]. Nevertheless, some authors reported that the use of starter cultures in sausages could slightly increase the content of biogenic amines [9]. However, recent studies have shown that autochthonous starter cultures may control the accumulation of biogenic amines in fermented meat products, while retaining their sensory characteristics [10,11].

Nowadays, the microorganisms mainly used as starters in the meat processing industry belong to four groups: (1) lactic acid bacteria (LAB) from the genus *Lactobacillus*, namely *L. sakei*, *L. plantarum* and *L. curvatus*; (2) Gram-positive catalase positive bacteria, such as coagulase-negative staphylococci (CNS), namely *S. xylosus* and *S. equorum*, and *Micrococcaceae*, namely *Kocuria* spp. [12,13,14]; (3) yeasts, such as *Debaromyces* spp. [15,16]; and (4) moulds of the genus *Penicillium* [17]. The first two groups are used for the inoculation of meat batters, the third group can be inoculated both in meat batters and on the surface of sausages, and the fourth group is used for the superficial inoculation of sausages [13,14,18,19].

The aim of the present study was to evaluate the effects of different autochthonous starter cultures on the safety and quality of *Painho da Beira Baixa*, a traditional Portuguese smoked fermented sausage produced on a small scale in a local small manufacturing unit.

## 2. Materials and Methods

### 2.1. Smoked Fermented Sausage Manufacturing and Sampling

*Painho da Beira Baixa*, a traditional smoked fermented sausage, was manufactured in a local factory using commercial white pig meat.

Pork meat trimmings (70% lean meat/30% fat) were mechanically cut into cubes of 35 to 45 mm and mixed with white wine (5.0% *v*/*v*), salt (2.5% *w*/*w*), red pepper (*Capsicum annuum* L.) paste (2.5% *w*/*w*), water (2.0% *v*/*v*), garlic (*Allium sativum* L.) paste (0.8% *w*/*w*), powder laurel (*Laurus nobilis* L.) (0.005% *w*/*w*), polyphosphates (0.06% *w*/*w*), nitrates (0.007% *w*/*w*), nitrites (0.003% *w*/*w*) and ascorbic acid (0.04% *w*/*w*).

Three independent batches of each treatment were prepared over time with a unit weight of approximately 150 kg (meat batter). Food grade dextrose (0.25%) was added to all treatments.

Starters and used concentrations were selected based on previous work [20,21] and were co-inoculated in the meat batters. In these earlier studies, different concentrations were tested in pure and mixed cultures. The best combinations of mixed culture/concentration were selected for further studies. Bacteria were inoculated at a concentration of 10^8^ cfu/g of meat batter, whereas the yeast was co-inoculated at a concentration of 10^6^ cfu/g of meat batter.

Five treatments were considered: 1—control (no starter cultures added); 2—*Staphylococcus equorum* S2M7/*Lactobacillus sakei* CV3C2; 3—*S. equorum* S2M7/*L. sakei* CV3C2/yeast 2RB4; 4—*Staphylococcus xylosus* CECT7057/*Lactobacillus sakei* CECT7056 and 5—*S. xylosus* CECT7057/*L. sakei* CECT7056/yeast 2RB4).

The strains *S. equorum* S2M7 and *L. sakei* CV3C2 were previously isolated and studied [22]. *S. xylosus* CECT7057 and *L. sakei* CECT7056 were isolated by Elias [21] and successfully used as starters in *Paio* from Alentejo. These two strains were latter subjected to a patent both in Portugal and in Spain.

Each meat batter was stored under controlled conditions at 5 °C and 90% relative humidity for 72 h for ripening purposes. Afterwards, the batter was stuffed into pork natural casings of 50–55 mm (pig’s large intestine). Salted natural casings were desalted as follows: casings were washed with running water, acetic acid (3.0% *v*/*v*) and commercial disinfectant (Desitripa, Formulab, Maia, Portugal) for 1 h. After draining, washed casings may be stored at 5 °C for a maximum of 24 h. Freshly stuffed sausages (weighting around 400–450 g/each) were left to ferment for 48 h at room temperature (between 15.1 and 22.3 °C), before they were smoked in a traditional smokehouse with natural smoke from *Quercus ilex* wood combustion. Sausages were continuously smoked (about 10 days) until they reached 38%–40% weight losses. Each sausage (end-product) weighted around 240–270 g. Temperature and relative humidity in the traditional smokehouse were monitored and registered with a data logger (Ebro EBI-20 THP, Ingolstadt, Germany), showing variations between 10.5 and 48.4 °C, and 31.7% and 84.8%, respectively.

Two separate sausages per treatment and per batch were collected throughout the curing process at four different steps: meat batter (immediately before stuffing), fermented sausage (48 h after stuffing and maintained at room temperature in the smokehouse antechamber), half-cured sausage (7 days after stuffing) and end-product (38%–40% weight losses).

PH, a_W_, microbiological parameters and contents of biogenic amines were determined at all curing steps. Evaluations of colour, texture profile analysis and sensory analysis were only performed in end-products.

### 2.2. Physicochemical Analyses

#### 2.2.1. Determination of pH and a_W_

Casings were removed, the sausages were minced, and pH was measured with a Crison 507 (Crison, Barcelona, Spain) pH-meter following the procedures described in ISO 2917 [23]. Water activity (a_W_) was determined at 25 °C with a hygrometer (Hygroskop Rotronic DT, Zurich, Switzerland) equipped with a WA-40 probe. Five replicates per sample were used.

#### 2.2.2. Colour

Colour was measured with a Konica Minolta CR-400 colorimeter (Konica Minolta Inc., Tokyo, Japan) on cross-sections immediately after cutting the sausages (to prevent colour degradation). Five replicates per sample were tested at room temperature (20 ± 1 °C). The CIELab chromatic coordinates lightness (L*), redness/greenness (a*) and yellowness/blueness (b*) were measured and chroma (C* = ((a*)^2^ + (b*)^2^)^0.5^) and hue angle (H° = arctan(b*/a*)) were calculated. All measurements were performed using the standard illuminant D65.

#### 2.2.3. Texture Profile Analysis (TPA)

Texture profile analysis (TPA) was performed at room temperature (20 ± 1 °C) using a Stable Micro System TA-Hdi (Stable Micro Systems, Godalming, England) following the procedures described before [24,25]. Cross-section samples (1 cm thick) were compressed twice in two consecutive cycles of 50% compression with 5 s breaks between cycles. A cylindrical flat-ended probe (with an area of 1 cm^2^) was actioned at a constant speed of 1mm s^−1^. Five replicates per sample were used. Hardness (N), adhesiveness (N·s^−1^), springiness, cohesiveness, resilience and chewiness (N) were calculated from the obtained force–time curves.

### 2.3. Microbiological Analyses

Microbiological analyses were performed following international standards and established procedures: mesophiles following ISO 4833-1 [26]; psychrotrophic microorganisms, ISO 17410 [27]; lactic acid bacteria (LAB) according to ISO 15214 [28], under anaerobiosis; staphylococci as described by Laranjo et al. [29]; yeasts and moulds, ISO 21527-2 [30]; enterobacteria following ISO 21528-2 [31]; and *Listeria monocytogenes* according to ISO 11290-2 [32]. For the detection of *Salmonella* spp., a VIDAS enzyme-linked fluorescent immunoassay (bioMérieux, Marcy-l’Étoile, France) was used. All positive results were confirmed according to ISO 6579-1 [33]. All microbiological analyses were performed in triplicate, and the results were expressed as log colony-forming units (cfu)/g, except for *L. monocytogenes* counts, which were reported as cfu/g.

### 2.4. Biogenic Amines Profile

Biogenic amine quantification was performed according to the experimental protocol described by Roseiro et al. [34]. Samples were homogenised using an IKA brand homogeniser, Ultra Turrax digital T25 model and 18G ST probe (Staufen im Breisgau, Germany) and extracted with 0.4 M perchloric acid (Panreac, Barcelona, Spain). 1,7-diaminoheptane (Merck, New Jersey, USA) was added as an internal standard. Sample extracts were derivatised with dansyl chloride (5 mg/mL acetone, *v*/*v*). Individual biogenic amines were separated and quantified in a Thermo Scientific Dionex High Performance Liquid Chromatography (HPLC) system, model Ultimate 3000 (Massachusetts, USA), equipped with a quaternary pump (HPLC Ultimate 3000 pump), automatic extractor (Ultimate 3000 Autosampler) and Array diode detector (DAD) (Ultimate 3000 RS Diode Array detector). The wavelength was adjusted to 254 nm, and an RP-18 reverse phase column (5 µm of 4.0 × 125 mm and 100 A˚) was used (Merck, NJ, USA). An elution program was carried out with a mixture of 0.1 M ammonium acetate (solvent A) and acetonitrile (solvent B). Quantification was done by external calibration, through the integration of areas obtained from a mixture of individual standards of known concentration.

All samples were extracted in duplicate, and each replicate was twofold derivatised. All replicas were injected twice. The following biogenic amines were quantified: tryptamine, β-phenylethylamine, putrescine, cadaverine, histamine, tyramine, spermidine and spermine. The content of each biogenic amine was expressed in mg/kg of fresh weight.

The content of vasoactive amines was calculated based on the sum of tryptamine, β-phenylethylamine, histamine and tyramine [35]. The total content of biogenic amines was calculated using the sum of all eight individual amines.

Chromatographic data were processed using the Chromeleon software version 6.8 (Thermo Scientific Dionex, MA, USA).

### 2.5. Sensory Analysis

Ten panellists were trained and selected following ISO 8586-1 [36]. Sensory evaluation took place in a specially prepared room in accordance with ISO 8589-1 [37]. Thirty minutes prior to each session, the sausages were sliced (3 mm thick) and the slices were randomly distributed in white dishes. Each dish was identified with a random three-digit number. Crackers and mineral water were provided, for the panellists to rinse their mouths between evaluations. The panellists were asked to evaluate products according to several attributes, using a quantitative descriptive analysis with a scale ranging from 0 to 100 corresponding to ‘‘no perception” or ‘‘maximum perception”. The evaluated attributes were colour intensity, off-colours, marbled, aroma intensity, off-aromas, hardness, fibrousness, succulence, flavour intensity, off-flavours, salt perception and overall appreciation. For hardness and salt perception, an optimum value of 50 was considered. Each panellist evaluated six samples per session.

### 2.6. Statistical Analysis

Data were analysed using the StatisticaTM v.8.0 software from Statsoft (StatSoftInc, 1984–2007, Tulsa, OK, USA). The elimination of outlier data was carried out according to the Grubbs test (α = 0.05).

Multi-factor analyses of variance or one-way ANOVAs were performed, and significantly different means were compared with the Tukey’s Honest Significant Difference (Tukey’s HSD) test (*p* < 0.05).

## 3. Results and Discussion

### 3.1. pH and a_W_

Table 1 summarizes the results for pH and a_W_ for the smoked fermented sausages subjected to different inoculation treatments. The values for both parameters gradually decreased throughout the curing process.

For pH, the control meat batter had a significantly higher mean value (5.78 ± 0.10) than all four inoculated meat batters, which showed no differences among them. The exact same is true for the fermented sausage, which means that keeping the sausages at temperatures close to 20 °C after stuffing allowed the microbiota to continue to multiply noticeably. This contributed to acid production, with the concomitant significant reduction of the mean pH values compared to the control. Franciosa et al. [1] and McLeod et al. [38] stated that the good adaptation of LAB to the conditions existing in the meat batters can contribute to their rapid multiplication. The fast proliferation of LAB is relevant in sausage manufacturing, as it leads to the metabolization of sugar with the consequent formation of organic acids, mainly lactic acid, which promotes a pH decrease [6,39]. Lowering the pH is essential as it contributes to the safety, development of a characteristic taste, colour, and aroma, as well as to the microbiological stability of the product. Regarding end-products, control sausages still had a significantly higher mean pH (5.12 ± 0.18), and there were no differences between inoculated sausages. It should be noted that inoculated sausages had mean values below 5.0, meaning that the pH dropped more slowly in the control group. These conclusions agree with those reported by others [8,18,40]. Our values were generally lower than those of other authors, in the meat batter as well as in end-products [12,18,19,40]. The addition of white wine in the formulation may have contributed to our lower pH values.

Concerning a_W_, there were no considerable differences between inoculated and control sausages. The a_W_ values obtained for end-products, although higher than those obtained by other authors [12,18,19,40], are within the acceptable range that does not compromise microbial stability or the overall quality of the sausages.

Our results are similar to those reported in the literature. Cruxen et al. [12] described a typical fermented meat sausage with a water activity (a_W_) of 0.92 and pH values between 4.8 and 5.5. Leistner and Rödel [41] reported that sausages with a pH below 5.2 and a_W_ below 0.95, or pH below 5.0 or a_W_ below 0.91, are stable food products that do not need refrigeration temperatures for preservation purposes. Hierro et al. [42] indicated values below 0.90 for a_W_ and 5.5 for pH as a good rule of thumb to maintain a high level of food hygiene in sausages.

### 3.2. Characterisation of the Microbiota throughout the Curing Process

The results for all microbiological analyses are summarised in Table 2. No Salmonella spp. was detected for any of the tested samples. This agrees with other authors stating that fermentation and curing are effective in the control of pathogens [43,44]. Hajmeer et al. [45] reported that smoking and the high temperatures associated with the smoking process had an antimicrobial effect on pathogenic microorganisms, such as *C. jejuni*, *E. coli* O157:H7, *L. monocytogenes*, *S. enterica* and *Y. enterocolitica*.

Significant differences were observed between control and inoculated meat batters for mesophiles, psychrotrophic microorganisms, LAB, staphylococci and yeasts, which could be related to the addition of starter cultures, as previously reported [8,40]. In fact, much higher LAB counts were observed in the inoculated meat batters. Initial LAB counts for inoculated meat batters were 3 to 4 log units higher than the control, reflecting the inoculation and, simultaneously, that the strains were able to rapidly multiply and adapt to the meat batter environment. Afterwards, LAB counts increased and remained high, for all treatments, up to the end-product. These steady LAB numbers throughout the curing are probably due to the decrease in fermentable carbohydrates [46] and water activity [40]. In line with the results obtained by other authors [40,47,48], they remained the dominant microbial group in end-products (7.32–8.61 log cfu/g). Our data reveal that the inoculated strains are well adapted to the meat batter. In fact, *L. sakei* is referred to as the prevalent species in fermented meat sausages due to its ability to adapt regarding processing conditions [49,50].

The enumeration of mesophilic microorganisms, the origin and behaviour of which are quite heterogeneous in the microbiota of sausages, is not considered a good indicator of food hygiene for this type of meat product, reflecting the generally high LAB counts observed in this study.

The counts of psychrotrophic microorganisms were slightly lower than those of mesophiles, which is in agreement with the results obtained by others [51,52,53] that reported psychrotrophic microorganisms to be usually lower than mesophiles in this type of sausage.

Staphylococci counts generally decreased from the meat batter and fermented sausage steps to the subsequent curing steps. Control meat batters showed a significantly lower mean value (3.30 ± 0.42 log cfu/g) than inoculated meat batters. Regarding fermented sausages, non-inoculated sausages had significantly lower staphylococci numbers when compared to the sausages inoculated with *S. xylosus* CECT7057/*L. sakei* CECT7056/yeast 2RB4. Considering half-cured sausages and end-products, there were no significant differences between treatments. The mean staphylococci count in end-products was between 3.51 and 4.19 log cfu/g. The decrease in staphylococci numbers was perhaps due to the poor competitiveness of staphylococci against LAB, which is associated with a pH decrease (reaching values below 5.0 in the present study), as already reported by other authors [8,54,55]. Furthermore, the two starter staphylococcal species have shown different performances. The *S. xylosus* strain is more persistent in meat batters and fermented sausages and performs better than the *S. equorum* strain in competition with the autochthonous microbiota. However, it seems to be strongly affected by smoke, since the staphylococci counts in the treatments with *S. xylosus* decreased by approximately 3 logs after the smoking step.

Regarding enterobacteria, no significant differences were observed in the meat batters. However, in the fermented sausages, significant differences were noticed, with inoculated sausages showing more enterobacteria. Concerning half-cured sausages, enterobacteria numbers were below the detection limit of the method for all treatments. Nevertheless, control end-products still showed a low number of enterobacteria (0.86 ± 1.33 log cfu/g), which may evidence the role of starters in eliminating/reducing enterobacteria. Other authors, such as Cadavez et al. [56], Lorenzo et al. [40] and Garcia Fontan et al. [57], obtained higher values in end-products (approximately 1 to 3 log cfu/g, depending on the treatment). It should be noted that Cadavez et al. [56] and Garcia Fontan et al. [57] also used traditional smoking to dehydrate the sausages, while Lorenzo et al. [40] did not.

Concerning yeast numbers, significant differences in the meat batter step were only observed between control and the meat batter inoculated with *S. xylosus* CECT7057/*L. sakei* CECT7056/yeast 2RB4, with the latter having higher counts. Regarding fermented sausages, control sausages had a significantly lower mean value than inoculated sausages. Both in half-cured sausages and end-products, no significant differences were observed between treatments, and the counts were generally low, with the highest numbers being for control sausages. Our yeast counts are much lower than those determined by Andrade et al. [58], who produced Spanish salchichón inoculated with several strains of D. hansenii at a concentration of approximately 10^6^ cells/g of meat batter; added dextrin, dextrose and lactose (in a commercial mix with unknown individual concentrations); and did not smoke the sausages. Those authors obtained relatively constant yeast numbers (3.0–6.0 log cfu/g) throughout the production process. It should be noted that the lowest counts were always obtained for control sausages. Our low yeast numbers may be due to smoking but also due to the fact that yeasts cannot compete with the microbiota present in the sausage environment, including the inoculated starter bacteria. In fact, Encinas et al. [59] reported lower values for smoked sausages compared to non-smoked ones. Leistner [60] also reported smoking to affect yeast counts but that factors such as time and temperature play a major role.

Moulds showed relatively low numbers throughout the curing process, with no differences between treatments at all curing steps, and with no moulds at all being detected in end-products. It is common that no moulds appear in end-product sausages. Casquete et al. [53]—in Spanish chorizo and salchichón, inoculated with *Pediococcus acidilactici* and *Staphylococcus vitulus* at concentrations of approximately 5 x 10^7^ cells/g of meat batter—started the curing process with mean values between 1.48 and 2.34 log cfu/g and ended up with mean values close to 2 log cfu/g in the end-products, with no significant differences between treatments. Alves et al. [61] obtained values close to 0.70 log cfu/g in non-inoculated Alentejano pig sausages and close to 1.5 log cfu/g for non-inoculated Iberian X Duroc pig sausages, for the end-products. Our results were even lower than those in the end-products, which may be an indicator of microbial stability, since high mould concentrations in end-products may lead to sensory impairments [17,62].

For *L. monocytogenes*, all results were below the detection limit of the method for all treatments, in both fermented and half-cured sausages. Moreover, *L. monocytogenes* was only detected in the non-inoculated meat batter (5.00 ± 8.37 cfu/g) and end-products (1.66 ± 4.08 cfu/g), which may indicate an antimicrobial action of the used starters against *L. monocytogenes*. However, it should be noted that all counts were lower than those specified in the current legislation: 100 cfu/g for ready-to-eat foods likely to allow the growth of *L. monocytogenes*, except those intended for infants and for specific medicinal purposes, according to Commission Regulation (EC) No 1441/2007 amending Regulation (EC) No 2073/2005 on microbiological criteria for foodstuffs [63]. Thus, starters seem to have had an important effect on the inhibition of this foodborne pathogen, as also reported by Cenci-Goga et al. [64].

### 3.3. Biogenic Amine Profile

Table 3 shows the content of biogenic amines for each treatment throughout the curing process. The main identified individual biogenic amines, in decreasing order of concentration, were tryptamine, spermine, putrescine and cadaverine. Histamine and β-phenylethylamine were only present at very low concentrations.

Table 3 shows that no differences were observed for histamine between curing steps and that the levels remained below 1.00 mg/kg for all treatments. Tyramine levels gradually increased throughout the curing process, with no significant differences between treatments. The mean values in end-products did not exceed 13.17 ± 5.59 mg/kg.

Regarding food safety, histamine and tyramine are the most toxic amines [65]. Daily maximum intakes of 50 mg of histamine and 600 mg of tyramine have been considered safe for healthy individuals [66]. However, Rauscher-Gabernig et al. [67] reported that the no-effect levels for alimentary histamine ranged from 6 to 25 mg/meal, whereas the ingestion of 70 mg/meal could trigger intoxication symptoms and 100 mg/meal, severe intoxications. Both tyramine and histamine had very low concentrations in the present work compared to those obtained by other authors [18,68,69].

The presence of natural polyamines, spermidine and spermine followed the pattern usually found in dry-cured sausages, with the greater prevalence of the last over the first [70,71,72,73,74]. Furthermore, Kalač and Krausová [75] report that meat and meat products generally have concentrations that rarely exceed 10 mg/kg for spermidine and that levels between 20 and 60 mg/kg are already considered high for spermine. However, Tasić et al. [73] have determined values of 101 mg/kg for spermine in non-inoculated Serbian sausages. Kalač [76] states that the presence of higher amounts of spermine is common in foods of animal origin, although the relative proportion between these two biogenic amines may vary depending on the type of raw materials used in formulating the sausages, which is corroborated by Stadnik and Dolatowski [77] and Ruiz-Capillas and Jiménez-Colmenero [78], who say that the levels of natural polyamines are generally reduced or maintained throughout the curing process. Nevertheless, Hernández-Jover et al. [79] reported that a reduction in spermine levels is more common, because it can be used by some microorganisms as a source of nitrogen.

For vasoactive amines (tryptamine, β-phenylethylamine, histamine and tyramine), the levels were somewhat lower in half-cured sausages, compared with at the previous curing steps. However, they slightly increased in end-products, even if with no significant differences. Control sausages had the highest mean value (147.26 ± 61.07 mg/kg), while sausages inoculated with *S. equorum* S2M7/L. *sakei* CV3C2/yeast 2RB4 had the lowest mean values, representing 28.96% less vasoactive amines. This corroborates the previous starter selection [22], showing that *S. equorum* S2M7 and *L. sakei* CV3C2 were low producers of biogenic amines. The addition of the yeast 2RB4 in co-culture with these two strains seems to reduce even further the production of biogenic amines, probably due to a better control of other bacterial strains able to produce biogenic amines.

Concerning the total content of biogenic amines, no significant differences were observed over time. However, control end-products showed the highest mean value (391.93 ± 184.28 mg/kg), which was significantly different from the mean value of sausages inoculated with *S. equorum* S2M7/*L. sakei* CV3C2/yeast 2RB4 (249.13 ± 29.69 mg/kg), representing a 36.44% reduction in total amines.

Regarding end-products, vasoactive amines were less than 200 mg/kg and total biogenic amines remained below 1000 mg/kg. These are positive results in terms of hygiene, since Tasić et al. [73] cite several authors and point to values above 1000 mg/kg as hazardous to consumers’ health. They also show, for some biogenic amines, the levels that are in compliance with good hygiene and manufacturing practices: 100 to 800 mg/kg for tyramine, 50 to 100 mg/kg for histamine, < 30 mg/kg for β-phenylethylamine, and values below 200 mg/kg for the sum of vasoactive amines. Vidal-Carou et al. [80] stress the importance of a careful raw material selection and production process to avoid or reduce the formation of biogenic amines. Silla Santos [81] indicate pH values between 4.0 and 5.5 as optimal for decarboxylation to occur. Others have reported the production of putrescine and cadaverine to be particularly associated with Gram-negative bacteria, mainly enterobacteria and pseudomonas [66,82,83]. In the present study, the pH mean value for the control end-products was significantly higher that for the inoculated sausages. Furthermore, the control sausages were the only ones to have enterobacteria (0.86 ± 1.33 log cfu/g) in end-products, which might be associated with their higher total amine content.

### 3.4. Colour

Table 4 shows no significant differences between treatments for any of the colour parameters studied. However, the sausages inoculated with *S. equorum* S2M7/*L. sakei* CV3C2, *S. equorum* S2M7/*L. sakei* CV3C2/yeast 2RB4 and *S. xylosus* CECT7057/*L. sakei* CECT7056 were slightly darker and redder (a*) than the others. Considering the values obtained for the C* coordinate, the inoculated sausages were slightly redder and with a stronger and brighter (more defined) colour.

Our results agree with those previously obtained by Essid and Hassouna [8], Casquete et al. [84] and Casaburi et al. [85], showing that starter cultures do not have a significant influence on colour parameters. Contrarily to our results, Lorenzo et al. [40] found that the use of starter cultures positively influenced the colour coordinates of sausages, while Casquete et al. [84] only identified an effect on the H° coordinate.

### 3.5. Texture Profile Analysis (TPA)

The results for the Texture Profile Analysis (TPA) are shown in Table 5. Significant differences between treatments were observed only for adhesiveness, which was significantly higher in sausages inoculated with *S. equorum* S2M7/*L. sakei* CV3C2 (−0.805 ± 0.632 N·s^−1^) and *S. equorum* S2M7/*L. sakei* CV3C2/yeast 2RB4, when compared to the non-inoculated sausages (−0.302 ± 0.293 N·s^−1^). Furthermore, the inoculated sausages were harder and showed higher chewiness values.

González-Fernández et al. [86] reported that pH values that reach the isoelectric point of proteins confer hardness and increase chewiness values due to the precipitation of proteins from sausages, a premise that the present work seems to corroborate.

Authors such as Lorenzo et al. [40] and Essid and Hassouna [8] concluded that inoculation with starter cultures did not have a significant effect on the textural characteristics of sausages, which were mainly influenced by ripening times. However, no comparison is possible with our results because we only carried out the determination of textural parameters in end-products. Nevertheless, Casquete et al. [84] inoculated *P. acidilactici* MS198 and *S. vitulus* RS34 at a concentration of approximately 5 × 10^7^ cells/g meat batter, with 2.5% of added sucrose, and observed a reduction in the hardness of the Spanish sausages, probably due to the proteolysis promoted by these lactic acid bacterial starters.

### 3.6. Sensory Analysis

Concerning sensory analysis (Table 6), the panellists did not detect significant differences for any of the evaluated attributes. Nevertheless, the panellists preferred control sausages in terms of colour intensity and aroma.

The sausages co-inoculated with the yeast strain 2RB4 were the only ones that did not show off-colours.

Regarding instrumental texture (Table 5), control sausages were less hard than inoculated sausages. However, the sensory panel considered control sausages slightly harder than inoculated sausages, although without significant differences. This may be due to some heterogeneity between the samples used for the two sets of analyses and might be related to common differences among samples (e.g., sausage diameter or poor fat distribution in the evaluated sausage slices) or a biased panellists’ assessment.

Concerning overall assessment, the sensory panel preferred the sausages inoculated with *S. equorum* S2M7/*L. sakei* CV3C2.

## 4. Conclusions

In the present study, the effect of autochthonous starter cultures on the safety and quality of a traditional Portuguese smoked fermented sausage, *Painho da Beira Baixa*, was evaluated. To our knowledge, this is the first comprehensive study on the quality and safety of this type of smoked fermented sausage from the central region of Portugal.

Starters did not have a noticeable effect on the reduction of a_W_, nor did they improve colour. However, significantly lower pH values were obtained for inoculated sausages. Furthermore, the effect of starters was observed in the absence of detectable enterobacteria and *L. monocytogenes* in all inoculated sausages. Moreover, sausages inoculated with *S. equorum* S2M7/*L. sakei* CV3C2/yeast 2RB4 promoted a reduction of 28.96% and 36.44%, respectively, for vasoactive and total amines. Nevertheless, regarding sensory evaluation, sausages inoculated with *S. equorum* S2M7/*L. sakei* CV3C2 had slightly higher scores in their sensory attributes. However, no influence on sensory attributes was observed in the co-inoculation with the yeast 2RB4, which was expected. This could probably be due to its lower ability to compete both with the autochthonous microbiota, as well as with the used bacterial starters.

In summary, the use of starter cultures did not compromise the quality of *Painho da Beira Baixa* regarding its sensory acceptability. Furthermore, some starters contributed to the safety of smoked fermented sausages, showing positive effects in reducing both the undesirable microbiota as well as the content of biogenic amines.

## Figures and Tables

**Table 1:** Effect of starter cultures on the pH and a_W_ of smoked fermented sausages.

Parameters	Treatment	Curing Steps			
		Meat Batter	Fermented Sausage	Half-Cured Sausage	End-Product
pH	1	5.78 ^A,a^ ± 0.10	5.59 ^B,a^ ± 0.03	5.08 ^C,a^ ± 0.07	5.12 ^C,a^ ± 0.18
	2	5.47 ^A,b^ ± 0.22	5.33 ^B,bc^ ± 0.11	4.97 ^C,b^ ± 0.04	4.92 ^C,b^ ± 0.08
	3	5.43 ^A,b^ ± 0.20	5.30 ^B,c^ ± 0.09	4.95 ^C,b^ ± 0.04	4.92 ^C,b^ ± 0.11
	4	5.47 ^A,b^ ± 0.23	5.30 ^B,bc^ ± 0.06	4.97 ^C,b^ ± 0.03	4.96 ^C,b^ ± 0.12
	5	5.48 ^A,b^ ± 0.21	5.35 ^B,b^ ± 0.07	4.98 ^C,b^ ± 0.05	4.95 ^C,b^ ± 0.08
a_W_	1	0.975 ^A,a^ ± 0.009	0.950 ^B,b^ ± 0.004	0.915 ^C,ab^ ± 0.009	0.876 ^D^ ± 0.006
	2	0.974 ^A,ab^ ± 0.012	0.963 ^B,a^ ± 0.012	0.914 ^C,ab^ ± 0.007	0.880 ^D^ ± 0.008
	3	0.968 ^A,bc^ ± 0.005	0.965 ^A,a^ ± 0.017	0.915 ^B,ab^ ± 0.013	0.879 ^C^ ± 0.003
	4	0.964 ^A,bc^ ± 0.008	0.962 ^A,a^ ± 0.011	0.919 ^B,a^ ± 0.005	0.881 ^C^ ± 0.015
	5	0.961 ^A,d^ ± 0.006	0.953 ^B,b^ ± 0.009	0.912 ^C,b^ ± 0.014	0.877 ^D^ ± 0.014

Data are expressed as means ± SD. 1—control; 2—*S. equorum* S2M7/*L. sakei* CV3C2; 3—*S. equorum* S2M7/*L. sakei* CV3C2/yeast 2RB4; 4—*S. xylosus* CECT7057/*L. sakei* CECT7056; 5—*S. xylosus* CECT7057/*L. sakei* CECT7056/yeast 2RB4. For the same treatment and in the same row, distinct capital letters (^A–D^) represent significantly different means (*p* < 0.05). For each curing step and in the same column, distinct lowercase letters (^a–d^) represent significantly different means (*p* < 0.05).

**Table 2:** Effect of starter cultures on the microbiological parameters of smoked fermented sausages.

Parameters	Treatment	Curing Steps			
		Meat Batter	Fermented Sausage	Half-Cured Sausage	End-Product
mesophiles	1	5.85 ^C,c^ ± 0.36	7.03 ^B,c^ ± 0.38	7.91 ^A^ ± 0.88	7.26 ^AB^ ± 0.15
	2	6.69 ^B,b^ ± 0.27	8.04 ^A,b^ ± 0.24	8.44 ^A^ ± 0.87	7.98 ^A^ ± 0.28
	3	7.20 ^B,ab^ ± 0.52	8.13 ^A,b^ ± 0.17	8.32 ^A^ ± 1.08	7.95 ^AB^ ± 0.21
	4	7.21 ^B,ab^ ± 0.33	8.12 ^AB,b^ ± 0.23	8.61 ^A^ ± 0.57	8.39 ^A^ ± 0.91
	5	7.27 ^B,a^ ± 0.22	9.07 ^A,a^ ± 0.73	8.14 ^AB^ ± 0.48	8.57 ^AB^ ± 1.64
psychrotrophic microorganisms	1	5.92 ^C,b^ ± 0.40	6.77 ^B,b^ ± 0.37	7.63 ^A^ ± 0.68	7.18 ^AB^ ± 0.27
	2	6.40 ^B,ab^ ± 0.52	8.11 ^A,a^ ± 0.33	8.43 ^A^ ± 0.91	7.82 ^A^ ± 0.48
	3	6.72 ^B,a^ ± 0.23	8.19 ^A,a^ ± 0.15	8.10 ^A^ ± 1.31	7.71 ^AB^ ± 0.36
	4	7.11 ^a^ ± 0.36	8.00 ^a^ ± 0.55	8.26 ± 0.87	8.17 ± 1.03
	5	7.02 ^B,a^ ± 0.45	8.43 ^A,a^ ± 0.46	7.60 ^AB^ ± 0.34	7.77 ^AB^ ± 0.75
LAB	1	2.91 ^B,b^ ± 1.56	7.15 ^A,c^ ± 0.39	8.06 ^A^ ± 0.93	7.32 ^A^ ± 0.23
	2	6.53 ^B,a^ ± 0.38	8.03 ^A,b^ ± 0.28	8.69 ^A^ ± 1.28	8.01 ^A^ ± 0.25
	3	7.00 ^B,a^ ± 0.58	8.03 ^AB,b^ ± 0.12	8.71 ^A^ ± 1.25	8.01 ^AB^ ± 0.18
	4	7.14 ^B,a^ ± 0.42	8,11 ^AB,ab^ ± 0.18	8.50 ^A^ ± 0.57	8.38 ^A^ ± 0.96
	5	7.30 ^a^ ± 0.51	8.78 ^a^ ± 0.75	7.85 ± 0.27	8.61 ± 1.66
staphylococci	1	3.30 ^c^ ± 0.42	4.10 ^b^ ± 0.51	3.66 ± 0.69	4.19 ± 1.01
	2	5.42 ^A,ab^ ± 0.51	5.51 ^A,b^ ± 0.61	3.86 ^B^ ± 1.05	4.12 ^AB^ ± 1.43
	3	5.04 ^b^ ± 1.11	5.42 ^b^ ± 1.03	4.15 ± 1.68	4.12 ± 1.25
	4	6.29 ^A,a^ ± 0.58	6.55 ^A,ab^ ± 0.50	3.78 ^B^ ± 0.49	3.55 ^B^ ± 0.44
	5	6.44 ^A,a^ ± 0.27	6.83 ^A,a^ ± 0.75	3.40 ^B^ ± 0.50	3.51 ^B^ ± 0.46
enterobacteria	1	4.43 ^A^ ± 0.50	2.58 ^B,b^ ± 0.28	<DL ^C^	0.86 ^C^ ± 1.33
	2	4.42 ^A^ ± 0.12	3.44 ^B,ab^ ± 0.39	<DL ^C^	<DL ^C^
	3	5.09 ^A^ ± 0.94	3.61 ^B,a^ ± 0.48	<DL ^C^	<DL ^C^
	4	5.12 ^A^ ± 0.77	4.06 ^A,a^ ± 0.90	<DL ^B^	<DL ^B^
	5	4.83 ^A^ ± 0.39	3.63 ^B,a^ ± 0.54	<DL ^C^	<DL ^C^
yeasts	1	3.59 ^A,b^ ± 0.46	0.92 ^B,b^ ± 1.02	0.46 ^B^ ± 1.13	0.88 ^B^ ± 1.42
	2	3.76 ^A,ab^ ± 0.42	3.54 ^A,a^ ± 0.40	<DL ^B^	0.74 ^B^ ± 1.15
	3	4.19 ^A,ab^ ± 0.09	3.93 ^A,a^ ± 0.20	<DL ^B^	0.17 ^B^ ± 0.41
	4	3.93 ^A,ab^ ± 0.36	3.52 ^B,a^ ± 0.26	<DL ^C^	<DL ^C^
	5	4.38 ^A,a^ ± 0.24	3.96 ^B,a^ ± 0.33	<DL ^C^	<DL ^C^
moulds	1	1.55 ^A^ ± 1.63	0.33 ^AB^ ± 0.54	<DL ^B^	<DL ^B^
	2	1.87 ^A^ ± 1.13	1.33 ^AB^ ± 1.28	<DL ^B^	<DL ^B^
	3	1.70 ± 1.97	<DL	<DL	<DL
	4	2.25 ^A^ ± 1.22	0.57 ^B^ ± 0,99	<DL ^B^	<DL ^B^
	5	1.64 ± 1.80	0.38 ± 0.94	<DL	<DL
*L. monocytogenes*	1	50.0 ± 83.7	<DL	<DL	16.7 ± 40.8
	2	<DL	<DL	<DL	<DL
	3	<DL	<DL	<DL	<DL
	4	<DL	<DL	<DL	<DL
	5	<DL	<DL	<DL	<DL

Data are expressed as means ± SD. < DL: below the detection limit of the corresponding analytical method (10 cfu/g). Results are expressed in log cfu/g. Results for *L. monocytogenes* are reported as cfu/g. 1—control; 2—*S. equorum* S2M7/*L. sakei* CV3C2; 3—*S. equorum* S2M7/*L. sakei* CV3C2/yeast 2RB4; 4—*S. xylosus* CECT7057/*L. sakei* CECT7056; 5—*S. xylosus* CECT7057/*L. sakei* CECT7056/yeast 2RB4. For the same treatment and in the same row, distinct capital letters (^A–C^) represent significantly different means (*p* < 0.05). For each curing step and in the same column, distinct lowercase letters (^a–c^) represent significantly different means (*p* < 0.05).

**Table 3:** Effect of starter cultures on the content in biogenic amines (mg/kg fresh weight) of smoked fermented sausages.

Parameters	Treatment	Curing Steps			
		Meat Batter	Fermented Sausage	Half-Cured Sausage	End-Product
tryptamine	1	138.70 ^ab^ ± 108.00	131.51 ± 60.31	74.17 ± 44.80	132.72 ^a^± 60.44
	2	112.17 ^b^ ± 68.07	119.88 ± 53.81	72.26 ± 41.23	104.34 ^ab^ ± 21.60
	3	158.05 ^A,ab^ ± 38.34	133.05 ^AB^ ± 64.67	93.09 ^BC^ ± 31.79	86.03 ^C,b^ ± 8.84
	4	208.21 ^A,a^ ± 86.79	107.23 ^B^ ± 38.17	85.21 ^B^ ± 33.02	122.39 ^B,ab^ ± 45.39
	5	140.52^A,ab^ ± 56.90	115.59 ^AB^ ± 53.22	83.32 ^B^ ± 28.42	103.00 ^AB,ab^ ± 34.36
β-phenylethylamine	1	5.00 ^A^ ± 2.37	4.27 ^AB^ ± 1.77	3.54 ^AB^ ± 0.54	3.27 ^B^ ± 0.44
	2	5.15 ± 3.10	3.97 ± 0.98	4.17 ± 0.98	3.77 ± 0.74
	3	9.81 ± 11.80	4.26 ± 2.30	3.93 ± 1.54	5.41 ± 3.99
	4	6.05 ^A^ ± 2.90	4.15 ^AB^ ± 1.00	3.66 ^B^ ± 0.66	4.21 ^AB^ ± 1.78
	5	7.15 ^A^ ± 4.51	3.95 ^B^ ± 1.11	3.20 ^B^ ± 0.58	4.05 ^B^ ±2.05
putrescine	1	7.79 ^C^ ± 2.75	8.61 ^BC^ ± 2.29	33.38 ^B^ ± 25.68	64.83 ^A,a^ ± 38.26
	2	10.78 ^B^ ± 4.23	10.63 ^B^ ± 10.72	34.79 ^A^ ± 26.31	31.00 ^AB,b^ ± 26.31
	3	10.96 ^B^ ± 3.31	9.84 ^B^ ± 3.79	29.38 ^A^ ± 21.33	15.94 ^B,b^ ± 9.49
	4	11.52 ± 4.18	10.37 ± 3.39	21.35 ± 12.40	21.73 ^b^ ± 17.16
	5	10.94 ± 5.41	9.81 ± 3.72	20.91 ± 16.21	19.58 ^b^ ± 13.08
cadaverine	1	18.90 ^B^ ± 5.38	20.53 ^B^ ± 2.82	1.88 ^B^ ± 5.28	27.58 ^A^ ± 9.67
	2	16.98 ^B^ ± 4.94	22.80 ^AB^ ± 9.66	21.16 ^AB^ ± 10.63	30.75 ^A^ ± 14.49
	3	19.95 ± 4.35	23.62 ± 14.76	19.72 ± 3.47	21.86 ± 10.60
	4	22.04 ± 11.48	19.32 ± 4.07	17.00 ± 5.10	22.48 ± 8.94
	5	28.53 ± 21.58	18.65 ± 4.14	17.73 ± 4.14	18.83 ± 8.28
histamine	1	0.32 ± 1.01	ND	0.28 ^a^ ± 0.54	0.80 ± 2.17
	2	0.55 ± 0.92	ND	ND ^b^	ND
	3	0.31 ± 0.76	ND	ND ^b^	ND
	4	ND	ND	ND ^b^	ND
	5	ND	ND	ND ^b^	ND
tyramine	1	1.33 ^C^ ± 0.85	3.18 ^C^ ± 1.90	6.90 ^B^ ± 4.16	10.46 ^A^ ± 2.37
	2	2.46 ^B^ ± 3.30	4.86 ^B^ ± 2.14	7.71 ^A^ ± 1.30	8.79 ^A^ ± 2.62
	3	1.77 ^C^ ± 0.84	3.91 ^BC^ ± 2.46	7.50 ^B^ ± 3.29	13.17 ^A^ ± 5.59
	4	3.05 ^B^ ± 3.84	4.62 ^B^ ± 2.15	7.25 ^AB^ ± 3.36	12.41 ^A^ ± 7.68
	5	1.16 ^C^ ± 1.01	4.33 ^B^ ± 1.57	6.69 ^B^ ± 2.71	10.63 ^A^ ± 3.09
spermidine	1	17.19 ^a^ ± 3.98	17.70 ± 4.09	17.54 ± 3.98	20.60 ^a^ ± 5.06
	2	16.03 ^ab^ ± 3.22	22.83 ± 16.45	16.95 ± 5.22	16.30 ^ab^ ± 3.91
	3	16.84 ^ab^ ± 3.10	12.69 ± 4.16	17.26 ± 3.11	18.75 ^ab^ ± 5.39
	4	13.76 ^ab^ ± 3.17	15.54 ± 2.40	17.20 ± 3.67	13.99 ^b^ ± 4.29
	5	11.74 ^b^ ± 5.73	14.99 ± 3.49	16.66 ± 4.81	13.86 ^b^ ± 2.91
spermine	1	100.50 ± 63.95	123.09 ± 53.48	121.76 ^b^ ± 43.20	131.66 ±97.55
	2	101.61 ^BC^ ± 59.05	155.32 ^A^ ± 46.65	129.38 ^AB,ab^ ± 32.05	75.14 ^C^ ± 12.01
	3	135.50 ^AB^ ± 51.05	123.25 ^BC^ ± 53.32	170.35 ^A,a^ ± 33.24	87.97 ^C^ ± 17.18
	4	123.45 ± 45.29	139.22 ± 33.36	131.42 ^ab^ ± 52.51	116.07 ± 52.17
	5	125.90 ^AB^ ± 47.61	154.76 ^A^ ± 46.47	130.61 ^AB,ab^ ± 29.46	105.07 ^B^ ± 31.85
vasoactive amines	1	145.35 ^ab^ ± 107.45	138.95 ± 59.91	84.89 ± 45.05	147.26 ± 61.07
	2	120.32 ^b^ ± 66.69	128.71 ± 51.95	84.13 ± 41.06	116.90 ± 22.14
	3	170.28 ^A,ab^ ± 41.20	141.22 ^AB^ ± 65.37	104.52 ^B^ ± 32.42	104.61 ^B^ ± 12.79
	4	217.31 ^A,a^ ± 89.43	116.00 ^B^ ± 37.38	96.13 ^B^ ± 32.53	139.00 ^B^ ± 44.52
	5	148.82 ^A,ab^ ± 58.82	123.87 ^AB^ ± 53.42	93.21 ^B^ ± 28.20	117.68 ^AB^ ± 36.43
total amines	1	289.73 ± 164.49	308.88 ± 72.10	275.45 ± 70.97	391.93 ^a^ ± 184.28
	2	265.72 ± 73.77	340.29 ± 73.77	286.50 ± 80.93	270.08 ^ab^ ± 39.99
	3	353.54 ^A^ ± 63.17	310.62 ^AB^ ± 94.20	341.23 ^A^ ± 68.58	249.13 ^B,b^ ± 29.69
	4	388.08 ^A^ ± 85.39	300.46 ^AB^ ± 66.94	283.10 ^B^ ± 68.44	313.28 ^AB,ab^ ± 112.86
	5	325.94 ± 110.90	322.09 ± 104.02	279.12 ± 44.16	275.03 ^ab^ ± 71.41

Data are expressed as means ± SD. N.D.—not detected; detection limit for histamine is 0.20 mg/kg. 1—control; 2—*S. equorum* S2M7/*L. sakei* CV3C2; 3—*S. equorum* S2M7/*L. sakei* CV3C2/yeast 2RB4; 4—*S. xylosus* CECT7057/*L. sakei* CECT7056; 5—*S. xylosus* CECT7057/*L. sakei* CECT7056/yeast 2RB4. For the same treatment and in the same row, distinct capital letters (^A–C^) represent significantly different means (*p* < 0.05). For each curing step and in the same column, distinct lowercase letters (^a,b^) represent significantly different means (*p* < 0.05).

**Table 4:** Effect of starter cultures on the colour parameters of smoked fermented sausages evaluated in end-products.

Treatment	Colour Parameters				
	L * (Lightness)	a * (Redness/Greenness)	b * (Yellowness/Blueness)	C* (Chroma)	H° (Hue Angle)
1	48.40 ± 5.52	14.96 ± 3.07	11.91 ± 3.44	19.23 ± 4.15	38.26 ± 6.01
2	46.70 ± 6.76	15.75 ± 3.11	12.50 ± 3.37	20.24 ± 3.94	38.23 ± 6.78
3	47.80 ± 5.59	15.08 ± 2.79	13.13 ± 3.30	20.15 ± 3.55	40.81 ± 6.95
4	46.98 ± 5.90	15.81 ± 2.57	12.75 ± 4.02	20.47 ± 4.03	38.13 ± 7.14
5	49.30 ± 5.83	14.70 ± 2.32	12.62 ± 3.26	19.58 ± 2.71	40.29 ± 8.71

Data are expressed as means ± SD. 1—control; 2—*S. equorum* S2M7/*L. sakei* CV3C2; 3—*S. equorum* S2M7/*L. sakei* CV3C2/yeast 2RB4; 4—*S. xylosus* CECT7057/*L. sakei* CECT7056; 5—*S. xylosus* CECT7057/*L. sakei* CECT7056/yeast 2RB4. In the same column, different letters represent significantly different means (*p* < 0.05).

**Table 5:** Effect of starter cultures on textural parameters of smoked fermented sausages evaluated in end-products.

Treatment	Texture Parameters					
	Hardness (N)	Adhesiveness (N·s^−1^)	Cohesiveness	Springiness	Resilience	Chewiness (N)
1	40.853 ± 17.730	−0.302 ^b^ ± 0.293	0.636 ± 0.055	0.855 ± 0.071	0.185 ± 0.033	22.665 ± 10.459
2	53.168 ± 18.532	−0.805 ^a^ ± 0.632	0.631 ± 0.053	0.853 ± 0.061	0.164 ± 0.030	28.754 ± 9.840
3	50.918 ± 18.852	−0.775 ^a^ ± 0.831	0.626 ± 0.041	0.864 ± 0.075	0.172 ± 0.028	27.892 ± 11.051
4	48.518 ± 14.997	−0.425 ^ab^ ± 0.232	0.627 ± 0.040	0.873 ± 0.060	0.177 ± 0.026	26.9892 ± 10.28
5	47.067 ± 21.018	−0.639 ^ab^ ± 0.691	0.629 ± 0.052	0.861 ± 0.069	0.181 ± 0.033	25.826 ± 11.744

Data are expressed as means ± SD. 1—control; 2—*S. equorum* S2M7/*L. sakei* CV3C2; 3—*S. equorum* S2M7/*L. sakei* CV3C2/yeast 2RB4; 4—*S. xylosus* CECT7057/*L. sakei* CECT7056; 5—*S. xylosus* CECT7057/*L. sakei* CECT7056/yeast 2RB4. In the same column, different letters (^a^ and ^b^) represent significantly different means (*p* < 0.05).

**Table 6:** Effect of starter cultures on the sensory attributes of smoked fermented sausages evaluated in end-products.

Treatment	Sensory Attributes											
	Colour Intensity	Off Colours	Marbled	Aroma Intensity	Off Aromas	Hardness	Fibrousness	Succulence	Flavour Intensity	Off Flavours	Salt Perception	Overall Appreciation
**1**	66 ± 9	0 ± 1	56 ± 1	70 ± 17	1 ± 5	58 ± 10	39 ± 27	61 ± 16	69 ± 11	3 ± 9	60 ± 8	62 ± 12
**2**	61 ± 11	0 ± 1	51 ± 8	65 ± 10	1 ± 5	56 ± 10	37 ± 23	58 ± 18	69 ± 10	4 ± 1	57 ± 7	64 ± 10
**3**	63 ± 12	0 ± 0	50 ± 5	63 ± 12	2 ± 5	57 ± 10	38 ± 27	56 ± 18	68 ± 11	4 ± 7	61 ± 7	58 ± 16
**4**	60 ±8	1 ± 3	53 ± 3	64 ± 11	2 ± 5	53 ± 10	40 ± 22	60 ± 18	64 ± 17	4 ± 7	59 ± 9	61 ± 15
**5**	61 ± 12	0 ± 0	55 ± 8	67 ± 10	2 ± 5	54 ± 10	38 ± 24	62 ± 15	70 ± 8	4 ± 7	58 ± 9	61 ± 16

Data are expressed as means ± SD. 1—control; 2—*S. equorum* S2M7/*L. sakei* CV3C2; 3—*S. equorum* S2M7/*L. sakei* CV3C2/yeast 2RB4; 4—*S. xylosus* CECT7057/*L. sakei* CECT7056; 5—*S. xylosus* CECT7057/*L. sakei* CECT7056/yeast 2RB4. In the same column, different letters represent significantly different means (*p* < 0.05).
